# Evaluating a Multi-pronged Initiative to Decrease Racial and Ethnic Inequities in the Identification of Maternal Substance Use: Successes, Challenges, and Lessons Learned

**DOI:** 10.21203/rs.3.rs-7602579/v1

**Published:** 2025-09-23

**Authors:** Melanie Besculides, Nicklas Sullivan Klepser, Parul Agarwal, Roland Clay Merchant, Yasmin L Hurd, Leah L Habersham

**Affiliations:** Icahn School of Medicine at Mount Sinai Department of Population Health Science and Policy; Icahn School of Medicine at Mount Sinai Department of Population Health Science and Policy; Icahn School of Medicine at Mount Sinai Department of Population Health Science and Policy; Icahn School of Medicine at Mount Sinai; Icahn School of Medicine at Mount Sinai Department of Psychiatry; Icahn School of Medicine at Mount Sinai Department of Psychiatry

## Abstract

Racial and ethnic disparities in prenatal substance use screening and subsequent referrals to child protective services (CPS) persist, despite similar rates of substance use across populations. The Basic Obstetric Addiction Screen (BOAS) Initiative was implemented in 2021 at two birthing facilities within a large medical system to address these inequities through a standardized, non-biased screening process. This study evaluated the initiative using a RE-AIM (Reach, Effectiveness, Adoption, Implementation, Maintenance) framework, analyzing electronic health record data, social work referrals, and qualitative feedback from healthcare providers and patients. Between January 2022 and November 2023, 99% of 21,342 pregnant patients were screened, with 0.45% screening positive. Among those screening positive, 71.9% were referred to social work. Qualitative analysis revealed six key themes: honesty requires fostering trust and addressing fear; BOAS implementation improved screening inequities, but they are still perceived to exist; BOAS is acceptable, EHR integration and a clinical champion facilitated its adoption; opinions vary on the best way to identify substance use; implementation was successful, but concerns exist; and many factors influence sustainability. Findings indicate that the BOAS Initiative is well-received by both patients and clinicians, with near-universal screening compliance and successful integration across both participating birthing facilities. The initiative demonstrated strong potential to reduce biased toxicology testing and unnecessary child CPS referrals.

## Introduction

Racial, ethnic, and socioeconomic inequities occur frequently in the United States (US) healthcare system. Such inequities are particularly amplified in the identification and care of prenatal patients with substance use disorders (SUDs). Despite comparable percentages of substance use among US prenatal populations, racial and ethnic minorities are subject to disproportionate biases, including targeted toxicology testing and the resultant increase in referrals to child protective services (CPS) agencies [1–8]. Despite these inequities, evidence is scarce on how healthcare systems can eliminate biased toxicology testing practices to address prenatal substance use equitably.

Between 2018 and 2020, advocacy groups unveiled biased toxicology testing practices within several New York City (NYC) healthcare systems [9–12]. As a result, the NYC Human Rights Commission began investigating hospital policies and practices believed to target Black and Hispanic mothers and infants in November 2020 [12]. In response, during January 2021, the Mount Sinai Health System’s (MSHS) Department of Obstetrics, Gynecology, and Reproductive Sciences (OBGYN) partnered with the Addiction Institute to create the Basic Obstetric Assessment Screen (BOAS) screening tool tailored for their maternal population. The tool was developed using existing validated tools: the 4Ps, the Substance Use Risk Profile Pregnancy Scale (SURPPS), and the Drug Screening Questionnaire (DAST) [13, 14] [15]. This screening tool coupled with a revised hospital policy on performing maternal and newborn toxicology testing during the prenatal and postpartum periods formed the *BOAS Initiative*. The purpose of the initiative was to identify maternal SUDs in a nonbiased manner. Patients who screened positive on BOAS would be referred to MSHS’s social workers and possibly linked to SUD care. A previous study using 2017–2022 MSHS data pre-initiative revealed non-White perinatal patients were toxicology tested more frequently with fewer positive results compared to their White counterparts. Disparities were notably eliminated post-implementation of the BOAS initiative [16].

This investigation assesses the implementation of the BOAS Initiative in MSHS Labor and Delivery (L&D) units, examining long-term sustainability, perceived disparities, trust in screening, implementation challenges, and opinions on substance use identification. We organized our assessment using the RE-AIM (Reach, Effectiveness, Adoption, Implementation, Maintenance) framework by examining the medical system’s electronic health record (EHR) data and social work referrals documentation, as well as through focus groups and individual interviews with physicians, social workers, and patients. The assessment findings have guided refinements of the BOAS Initiative and its evaluation metrics and may assist others implementing prenatal substance use screening tools and policies.

## Materials and Methods

### Study design and location

This investigation used multiple methods to examine the impact of the BOAS Initiative after its implementation in 2021 at the two Mount Sinai facilities with birthing capacity (Mount Sinai Hospital and Mount Sinai West). Quantitative data is from these facilities, and qualitative data is from workers or patients at the facilities. Qualitative reporting follows the Consolidated Criteria for Reporting Qualitative Research (COREQ) standards [17].

#### RE-AIM (Reach, Effectiveness, Adoption, Implementation, Maintenance) framework

The RE-AIM framework was used to organize the evaluation [18]. RE-AIM is a well-established, comprehensive evaluation framework for assessing the implementation of public health interventions. RE-AIM domains were addressed by examining the medical system’s EHR data and social work referral documentation, as well as through focus groups and individual interviews of physicians, social workers and patients. *Reach* was measured by the percentage of patients admitted to L&D who had a BOAS score entered. Effectiveness was evaluated by analyzing the proportion of patients with a positive BOAS screen, which aims to reflect the true prevalence of substance use in prenatal populations. Additionally, effectiveness was assessed by examining documentation of indications for social work referrals to CPS, specifically the New York City Administration for Children’s Services and the Department of Health and Mental Hygiene, to evaluate alignment with the initiative’s objective of eliminating referrals based solely on parental substance use [19]. *Adoption* was measured by the number of sites which implemented the initiative among the birthing centers within the MSHS. *Implementation* was assessed by the percentage of patients with positive BOAS screens referred to social work. *Maintenance* was only assessed during the focus groups and individual interviews.

#### Quantitative data: EHR and social work referrals

BOAS score entries were evaluated during L&D admissions from January 1, 2022 (to allow time for uptake of the screening tool in L&D) to November 30, 2023. Social work referrals to CPS were recorded from January 1, 2021 to December 31, 2023. CPS referral data were only recorded and available from Mount Sinai Hospital, not Mount Sinai West.

#### Qualitative data: Physician, social worker and patient focus groups and individual interviews

Focus group and interview participants were recruited from the Departments of OBGYN, Neonatology and the Department of Psychiatry’s Addiction Institute of Mount Sinai (AIMS). Attending physicians were purposefully sampled and included BOAS initiative policy developers, implementors, and utilizers. Social workers conducting BOAS screening in the prenatal setting were included. Physicians and social workers were recruited via email. Patients eligible for participation were pregnant or within two years postpartum, 18–50 years-old, English speaking, and receiving care through either the AIMS or the Department of OBGYN. Individuals with and without a history of SUD were included. Patients were recruited through fliers posted in the outpatient clinic or during hospitalizations on the L&D and postpartum units. Participants completed a REDcap survey eliciting demographic characteristics.

Focus groups and interviews were conducted via HIPAA-compliant Zoom^™^ and recorded with permission. Focus groups and interviews were conducted in English using a semi-structured guide (Appendix) from November 2023 to May 2024. LH (female physician with no hierarchical power over those interviewed) conducted the physician interviews. A qualitative consultant (female licensed therapist not involved in the care of patients interviewed and without personal/professional relationships with social workers interviewed) conducted social worker and patient focus groups and interviews. Interviews were conducted when focus groups were not feasible for social workers or patients. Focus groups lasted approximately 1 hour and interviews approximately 30 minutes. Knowledge saturation was discussed bi-weekly and recruitment ceased when saturation was reached for each group. Participants were compensated with a gift card for their time.

#### Quantitative data statistical analysis

Quantitative data were summarized using descriptive statistics. Screening rates and patient referrals were evaluated across the system. Analyses were conducted using Statistical Analysis System (SAS^®^) OnDemand for Academics (SAS Institute Inc., Cary, NC, USA) [20].

### Qualitative data analysis

Recordings were professionally transcribed, checked for accuracy, and imported into Dedoose 9.0 for coding and analysis [21]. Two researchers (MB, NK) independently coded each transcript, with weekly meetings to resolve differences and discuss emerging codes. The final codebook (Supplemental Table 1) was then applied to all previously coded transcripts and thematic analysis was conducted. Themes and subthemes were categorized according to RE-AIM domains and illustrative quotes were chosen.

#### Standard protocol approvals, registrations, and patient consents

Standard protocol approvals, registrations, and patient consents

The Icahn School of Medicine at Mount Sinai Institutional Review Board approved the study (22–01190). Informed consent was obtained from each qualitative participant prior to data collection.

## Results

### Focus group and interview participant characteristics

**Figure 1:**
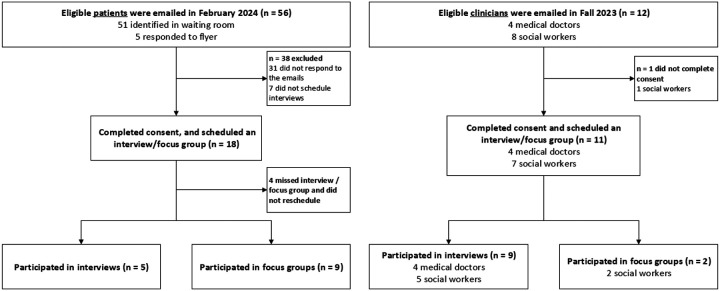
Participant Flowchart for Interviews and Focus Groups

Figure 1 provides a participant recruitment flow diagram. Approximately 92% of physicians and social workers and 32% of patients contacted agreed to participate in the study. Fourteen patients, seven social workers, and four physicians attended focus groups or interviews (n = 25). Table 1 illustrates physician and social worker characteristics and Table 2 patients.

### RE-AIM

Results are presented by RE-AIM domain. Table 3 summarizes quantitative data and qualitative insights. Table 4 lists themes and subthemes and illustrative quotes supporting the themes.

### REACH

#### Compliance with BOAS screening at L&D admission

Of 21,342 patients admitted to L&D, 99.2% had a BOAS score entered in their EHR.

#### Theme 1: Honesty Requires Fostering Trust and Addressing Fear

Full participation requires patient honesty during the BOAS screening, and this is affected by many factors.

##### Subtheme 1a: Honesty requires trust in and comfort with provider.

Patients, physicians and social workers emphasized building trust and comfort to encourage transparency about substance use. Some patients recalled discomfort when asked private questions by social workers in shared office spaces. Physicians and social workers stressed building rapport with patients and working to overcome fear of the medical system originating from past interactions.

Patients reported feeling more comfortable with providers who reflected their age, race, and language spoken. Patients also acknowledged that a provider’s race and age may affect care, with one noting different care experiences with White providers than providers of color. Patients also had conflicting opinions on the impact of provider age on a provider’s likelihood to assess patients using toxicology tests.

##### Subtheme 1b: Honesty requires the right people asking the right questions in the right manner.

Some patients suggested that providers need more time to ask screening questions. Patients also recommended questions be posed conversationally by an obstetrician or known provider. Several patients noted that questions containing the words ‘problem’ or ‘disorder’ might discourage honest responses. Some disliked questions about the substance use of loved ones; stating a desire to not be judged on the behavior of others. Further suggestions included assessing timing (e.g., Have you felt the need to cut back? When?) and asking if one was being forced to use substances.

##### Subtheme 1c. Honesty requires wanting help

Patients noted that openness about drug use relies on their desire for assistance and their recognition of their use as a problem. One social worker noted that the screening tool could allow patients to flag themselves as having a substance use disorder.

##### Subtheme 1d: Fear of consequences remains

Patients and social workers acknowledged that fear of consequences, such as medical record documentation, provider judgment, involvement of CPS, or forced treatment, may still limit some pregnant women’s honesty in disclosing substance use.

### EFFECTIVENESS

#### Positivity Rate of BOAS Screening at L&D Admission

Of the 21,168 admissions to L&D with a BOAS score entered, 0.45% screened positive.

#### Documentation of indications for social work referrals to CPS

In 2021 (the first year of implementation) there were no social work referrals to CPS for substance use alone. In 2022, two referrals were made for substance use alone. In 2023, while no referrals were made for substance use alone, there were two referrals for substance use with an additional parental concern (e.g., inadequate guardianship).

#### Theme 2: BOAS Implementation Improved Toxicology Testing Inequities, but they are Still Perceived to Exist

Physicians and social workers remarked that toxicology testing and referral to CPS declined dramatically after the implementation of the BOAS initiative; providers did not test for cannabis and tested only when patients had medical indications. Physicians also stated that they did not refer to CPS for substance use alone. What constitutes a medical indication for toxicology testing is somewhat ambiguous. Providers noted a heightened awareness about toxicology testing equality, acknowledged that testing is not perfectly equitable, and recognized that stigma remains. Patients believed that non-White individuals were targeted by toxicology testing, and that providers are less trusting of their substance use screening responses.

Providers acknowledged other factors that reduced disparities including increased provider awareness and education, equity-driven resident physicians, and New York State’s cannabis legalization in 2021. A physician remarked that the policy has not increased clinician awareness of the importance of identifying substance use in all patients.

### ADOPTION

#### Implementation of the BOAS initiative among the eligible birthing centers

Both MSHS facilities with birthing capacity adopted the BOAS initiative.

#### Theme 3: The BOAS Tool is Acceptable, and EHR Integration and a Clinical Champion Facilitated its Adoption

Providers found the BOAS tool and the broader initiative acceptable, appreciating its ease-of-use and integration into the EHR. Providers emphasized that a physician champion ensured integration into the clinical workflow. Patients who remembered being asked “BOAS-like” screening questions found them acceptable and appreciated the tool’s brevity.

#### Theme 4: Opinions Vary on the Best Way to Identify Substance Use

Four preferred ways of identifying substance use were mentioned: conversation, universal screening, chart review, and universal toxicology testing. The most common opinion was that an open conversation in a safe space, built on established trust, was the best strategy. One physician emphasized the value of universal screening to directly address substance use. One social worker thought a chart review coupled with a toxicology test was most effective.

Despite varying opinions on how to best identify substance use, most clinicians agreed that the BOAS initiative policy was followed, and infrequent deviators were re-educated. Although monitoring individual provider’s toxicology orders is not routine, providers did not believe people deviated repeatedly.

### IMPLEMENTATION

#### Referrals to social work for positive BOAS screens

Among those who were screened with BOAS and had a positive result, 72% had accompanying referrals to social work. Rates of social work referral did not vary significantly by facility (p = 0.17).

#### Theme 5: Implementation was Successful, but Concerns Exist

Providers reported that implementation overall was successful, but they had some concerns about resource limitations, the quality and availability of referral programs, and communication among providers. Physicians specifically raised concerns about a lack of physicians specialized in maternal substance use and the inconvenient location of the in-system SUD rehabilitation facility. In addition, social workers identified the need for continued BOAS training and workflow integration that emphasizes when plans for safe care are necessary. Physicians observed that not all patients who should have social work referrals based on their BOAS scores receive them. Social workers expressed concerns regarding the quality of treatment programs that are available for patient referrals. A social worker stated that providing safe and effective SUD treatment programs would be the most beneficial step for patients.

### MAINTENANCE

#### Theme 6: Many Factors Influence Sustainability

Providers thought OBGYN residents helped drive the change in substance use by re-framing from abuse to a disorder. Adapting BOAS from a paper screening tool to EHR integration, noted in ADOPTION, increased ease of use and led to maintenance. The limited number of clinicians trained to manage SUDs among pregnant women could also affect program maintenance as concern was raised over the consequences if these individuals left the institution.

## Discussion

The BOAS Initiative was successfully implemented in a large, urban health system serving a diverse population of patients. The implementation at both target sites and the 99% screening rate indicates a high adoption. The initiative was considered acceptable despite varying opinions on substance use screening, and suggestions for tool improvement. Furthermore, adoption was facilitated by the tool’s ease-of-use, EHR integration, and the leadership of a clinical champion.

Less than 1% of BOAS scores entered were positive. In the literature, self-reported substance use during pregnancy ranges from 0.3% to 20%, depending upon substance and screening tool used [13, 22–26]. The National Survey on Drug Use and Health (NSDUH) reports overall substance use during pregnancy to be 5.1%, lower than the general adult population [27]. The low screen positive rate among our patients suggests a lack of patient disclosure. Research suggests that racial disparities in toxicology testing among patients who disclose substance use exist, with Black patients more likely to be tested than White [28]. CPS reporting is more likely to occur for Black than White patients, despite equal use of substances [29]. These key issues should be kept at the forefront of considerations while attempts toward improved disclosure are pursued. Providers and patients emphasized that patient-provider relationships which address fear of stigma and consequences facilitate honest reporting. Both acknowledged that readiness for change varies, potentially impacting honesty in substance use screenings.

### Implications for Practice

Providers noted a drastic decline in toxicology testing after initiative implementation, consistent with previously presented declines in testing [16]. Clinicians mentioned they refrain from testing based solely on suspicion of cannabis use and only test when medically indicated. They emphasized that CPS referrals are not made for substance use alone, a finding confirmed by our review of referrals from Mount Sinai Hospital. There were no referrals for substance use in the first year of the initiative alone or in combination with other reasons followed by a leveling off in subsequent years. This “hype cycle” has been reported previously for innovation uptake [30]. We did not collect CPS reporting data prior to the initiatives’ implementation in 2021 for comparison. However, data from the New York City Administration for Children’s Services (ACS) shows an overall decrease, between 2017 and 2022, in hospital reports submitted citywide to the state central registry involving allegations of parental substance misuse with infants less than one month old [31]. Despite the BOAS Initiatives’ effectiveness, many noted that toxicology testing is still not completely equitable, suggesting the need for ongoing training.

### Implications for Research

Although the majority (72%) of those who screened positive on the BOAS were referred to social work, future research should evaluate why some patients were not referred and identify barriers to referral including resource limitations and training needs. Barriers to successful screening and intervention for smoking and alcohol programs have been shown in the literature [32]. Tracking the outcomes of referrals and variation in outcomes is another potential area for future research.

The findings around the MAINTENANCE domain are encouraging: the Initiative remains in place, the tool was integrated into the EHR, and a reported change in provider mindset surrounding substance use has occurred. Future work will examine strategies to sustain the Initiative including resident education.

### Strengths and Limitations

One strength is that the study population was racially and ethnically diverse and included those with SUD, which adds significantly to the literature. One limitation is that the study was conducted in a single health system; thus, the results may not be externally valid to other settings. Another limitation is the low response rate among patients, pregnant and postpartum women. Patients with SUD have many life demands and challenges that influence their willingness to participate in research studies. Also, only one facility tracked referrals to CPS. Finally, given the timing of the NYC Human Rights Commission investigation beginning in November 2020 and New York’s cannabis legalization in March 2021, outcomes cannot be solely attributed to the BOAS Initiative. However, the initiative was well-received by providers and contributed to reducing disparities in substance use screening.

## Conclusions

The BOAS initiative was effectively implemented to enhance the identification and management of substance use within prenatal care settings, demonstrating its practical impact on maternal health services. It was accepted by patients and providers, with some challenges for effectiveness and sustainability remaining. Future initiatives should consider how to increase patient disclosure, program sustainability, and adaptations needed to scale across varied healthcare settings.

## Supplementary Material

Supplementary Files

This is a list of supplementary files associated with this preprint. Click to download.

- AppendixQualitativeGuideQuestions.docx
- BOASISSMCOREQChecklistfinalJUH2.pdf
- SupplementalTable1CodebookforQualitativeData.pdf

## Figures and Tables

**Table 1:** Characteristics of clinician interview and focus group participants

Demographic	Clinicians *n*=10
**Age (years)**	
Mean (SD)	40.1 (12.0)
Range (Min - Max)	33 (27–60)
**Gender**	
Male	1 (10%)
Female	9 (90%)
**Race**	
White	5 (50%)
Black or African American	2 (20%)
Asian	1 (10%)
More Than One Race	2 (20%)
**Ethnicity**	
Hispanic or Latino	2 (20%)
NOT Hispanic or Latino	8 (80%)
**Years in Service (years)**	
Mean (SD)	14 (13.5)
Range (Min - Max)	35 (1–36)

Values for categorical variables are shown as count (%).

**Table 2:** Characteristics of patient interview and focus group participants

Demographic	Patients *n*=14
**Age (years)**	
Mean (SD)	28.2 (4.4)
Range (Min - Max)	17 (20–37)
**Race**	
White	4 (29%)
Black or African American	7 (50%)
More Than One Race	2 (14%)
Unknown/ Not Reported	1 (7%)
**Ethnicity**	
Hispanic or Latino	5 (37%)
NOT Hispanic or Latino	7 (50%)
Decline to Answer	1 (7%)
Unknown	1 (7%)
**Employment status**	
Employed	6 (43%)
Unemployed	8 (57%)
**Education**	
High School Diploma/GED	6 (43%)
Some College	4 (29%)
College Degree	3 (21%)
Graduate Degree	1 (7%)
**History of substance use disorder**	
Yes	3 (21%)
No	11 (79%)
**Parenting Status**	
Currently pregnant	10 (71%)
Less than one year postpartum	4 (29%)
**Insurance type**	
Commercial Insurance	1 (7%)
Medicaid	12 (86%)
Medicare	1 (7%)

Abbreviations: GED, General Educational Development Test; Values for categorical variables are shown as count (%).

**Table 3:** Mixed-Methods Findings by RE-AIM Dimensions

RE-AIM Domains	Quantitative Data Data Source and Time Period Measure(s)	Qualitative Measures Findings from Interviews and Focus Groups; Nov. 2023 to May 2024
Reach	EHR; 2022–2023 99.2% of patients admitted to L&D had a BOAS score recorded; 0.45% screened positive	Full participation requires patient honesty on the BOAS screening
Effectiveness	Internal documentation of SW referrals; 2021–2023 0 reports to CPS for substance use alone in 2021, 2 reports for substance use alone in 2022, and 2 reports for substance use with another issue in 2023	Physicians noticed decrease in testing and referrals, ambiguity exists about medical indications for toxicology testing; patients felt non-White individuals were targeted by toxicology testing and that providers distrusted their screening responses
Adoption	EHR; 2021 Both MSHS facilities with birthing capacity adopted the BOAS initiative	EHR integration and physician champion helped adoption, continued training needed, variation in preferred method of identifying substance use; patients found screening acceptable
Implementation	EHR; 2022–2023 71.9% of patients who screened positive were referred to social work	Concerns about resource limitations, referral programs, and communication among providers
Maintenance	NA	Initiative ongoing, EHR integration, changed provider mindset on substance use

Abbreviations: BOAS, Basic Obstetric Addiction Screen; CPS, Child Protective Services; EHR, Electronic Health Record; L&D, Labor and Delivery; MSHS, Mount Sinai Health System; SW, social worker

**Table 4:** Themes and Illustrative Quotes

THEMES	QUOTES
1. Honesty Requires Fostering Trust and Addressing Fear	**Subtheme 1a: Honesty requires trust in and comfort with the provider** “I just find it a little bit uncomfortable to be truthful answering the questions, especially just with a social worker because with the provider, with the doctor, you have the privacy to it. And it is someone that you’re going to keep seeing regularly.” [Patient FG 11,12,13] “I still think there’s a lot of stigma around drug use and I don’t think that patients are necessarily forthcoming, especially if they don’t have an established rapport with the person that they’re seeing as a provider. “ [MD 1130] “I know that there’s a lot of bias from medical providers, like doctors and nurses. They might see a White patient versus a Black or Hispanic patient, might be treated differently by that White provider. The care I’ve received from people of color has been different than the care I’ve received from White providers..” [Patient FG 1,2] **Subtheme 1b: Honesty requires the right people asking the right questions in the right manner** “… I think that those questions are good, but I also think that they could be worded in a way that invites a person who is really heavily addicted on substances to not feel like they’re being judged….the word ‘problem’, did you ever have a problem? Because some people that are in the middle of addiction, they might not consider it as a problem. They might be like, well I don’t have a problem. This is fine for me and it’s working for me, stuff like that. Or some people don’t like the word disorder for whatever reason. And sometimes people who deal with alcoholism, they don’t see it as an issue. [Patient FG 1,2] “I want to get to the family history, like what do you all mean? You all trying to, because my family had problems with that, you all trying to put that on me, trying to make it seem like I do, too? Because if you answer that—and you say family history problems with drugs or alcohol, some of my family members do have a problem with drugs and alcohol, so what you all trying to say? Like you’re trying to bring it to me? Like I don’t understand that… you have nothing to do with it…. The same thing with your partner, like if you had a partner and he’s a drug user, that doesn’t mean you are a drug user. [Patient FG 11, 12, 13] **Subtheme 1c: Honesty requires wanting help** “You’re gonna say yes to stuff if you want help for it and if you don’t want help and if you don’t feel like you got a problem with it, you just going to check no.” [Patient FG 11,12,13] **Subtheme 1d: Fear of consequences remains** “But in those conversations, some people were like, don’t tell your doctors, stuff like that. So I think there is some fear. Some people might lie, essentially. Because they’re scared of what might happen, they’re going to be judged by the providers, or maybe it’ll go on their medical record” [Patient 1, 2] Some may not for fear that they’re going to be judged, and that we’re going to involve ACS. I think that’s why most patients aren’t truthful. [SW 6,7]
2. BOAS Implementation Improved Screening Disparities but They are Still Perceived to Exist	“No one asks me to put in a urine toxicology anymore and I don’t have to argue and fight with them about it anymore…And when I started at Mount Sinai, I was asked like every day, can you put in a urine toxicology for me, right and I don’t see that at all anymore. [MD 1116] “I think it’s better. I don’t think we’ve reached a point where we can say there are no inequities. Again, because I really question how the nurses are screening, how are they asking the questions, if they’re asking all of the questions of everyone. But in general, I think that there has been a huge improvement, because there is a process now, by which we ask everyone. So if that process is followed, there shouldn’t be any inequities, because we’re not selecting who gets tested or not. [SW 6,7] So it’s like of course, you know your income play a part in it. If you, especially if you’re going in looking one type of way, yeah they’re going to automatically, especially if you’re going in looking rough, they’re gonna, of course you’re doing something, you know. So it’s like yeah they take all of that into account. And don’t let you be a minority, black or Latino or anything. You got to have done something. There is no oh no, I’ve never smoked. Oh I’ve never drank. Never? Not even once? And then they start asking you like that. Not even once? No, not even at a party? It’s like okay. So yeah it matters. It definitely matters.” [Patient FG 11,12,13]
3. The BOAS Tool is Acceptable and EHR Integration and a Clinical Champion Facilitated its Adoption	“I think it was kind of just put into Epic and it’s like a hard stop when nurses can’t, they have to complete it so it usually does get completed. [MD 1128] “I feel those questions are like the best questions to ask, as far as substance abuse and pregnancy, because they’re straight to the point, and they’re the most critical to a pregnancy, the early days of a pregnancy. It’s just straight to the point” [Patient 5] “I feel like it’s good. It’s, I feel like, again, you can get an idea from the questions that’s already on there. I don’t feel like more needs to be added because it could possibly make you a little uncomfortable because they are repetitive but it’s not too overwhelming and it’s the right questions to be asked. And it could possibly help, you know further down the line.” [Patient 10]
4. Opinions Vary on the Best Way to identify Drug Use	“The best way in my opinion is to just have an open dialogue with the patient and to make sure that the patient feels like they’re in a safe space so that they’ll be forthcoming and not feel like they’re being asked about drug use so that there could be punitive measures. It’s just so that we could determine whether or not they need additional services or counseling and not to be used as a punitive measure”. [MD 1130] “A urine sample is the best way to find the accurate information. Not everyone is truthful, unfortunately, about their usage. In order to help them and give them resources, it’s better to find out early on. Follow up testings and things like that.” [SW1] “Yeah. So we do a chart review to sort of get an idea of their history. And from there, we can see if there’s been any substance use present. And that’s a good indicator. That’s probably the best indicator, especially since we’re meeting these patients for the first time.” [SW2]
5. Implementation was Successful but Concerns Exist	“I don’t think that every nurse does her due diligence. I think sometimes questions are skipped or it’s done quickly – I just don’t trust that they’re always following it, and I think it’s because of bias. I think if it’s a White, middle class or wealthy woman, I think they might just assume that there’s no drug use and may not even ask. [SW FG 6,7] “I think what’s hard is that patients are very, and I don’t blame them, but the programs that we do have at our hand to refer, they’re not the best. So, you know people still use there or there’s just a lot of not great things going on within these programs. So I think what would be best is more actually helpful, safe, good programming. But again, there’s only so much we can do. But I do think it’s been stronger, at least from when I’ve had conversations with other providers of, that when we’re on top of this, then we had and I think that’s also from Dr. X, like her amazing work here.” [SW 5]
6. Many Factors Influence Sustainability	“I think in general the whole conversation has changed about substance use in pregnancy and I think that’s really great.” [MD 1116] “And it was also a push by the residents to be like this is super unfair and super racist and super classist and we need to stop doing this, right.” [MD 1116] “I mean I’ve only ever encountered I think two physicians who helped…. So I always wonder if we lose one or both of you will we still have those same resources? Are there other people to fill in those gaps? “ [MD 1128]

Abbreviations: FG, focus group; MD, Doctor of Medicine; SW, social worker

## Data Availability

The datasets generated during and/or analyzed during the current study are available from the corresponding author on reasonable request.
